# “I´m the one who has written this”: reciprocity in writing courses for older adults in Norway

**DOI:** 10.1080/17482631.2019.1650586

**Published:** 2019-08-07

**Authors:** Olga V. Lehmann, Svend Brinkmann

**Affiliations:** a Department of Mental Health, NTNU-Norwegian University of Science and Technology, Trondheim, Norway; b Department of Communication and Psychology, Aalborg University, Aalborg, Denmark

**Keywords:** Writing course, journaling, older adults, aging, self-exploration, otherness, togetherness, existential meaning, meaning-making, motivation

## Abstract

**Purpose**: The aim of this article is to explore, theoretically and empirically, the reciprocity of care afforded by writing courses as community interventions for older adults.

**Methods**: We narratively analyzed 209 excerpts of the anthology “I´m the one who has written this” written by teachers and participants of courses organized by the Church City Mission in Norway.

**Results**: The reciprocity that appeared in the writing courses is grounded in the sense of vulnerability that both teachers and participants embraced, and that is experienced in three main relational movements that these writing courses convey: self-exploration, otherness and togetherness. In addition, the data suggests that these courses promote affective processing and existential meaning-making, motivation, as well as improvements of memory and attention. However, more research is needed to confirm these preliminary findings, and their possible effects in older adults with and without symptoms of dementia.

**Conclusion**: Even though these writing courses for older adults are not explicitly therapeutic, they can have therapeutic effects, given the reciprocity afforded in these cultural community interventions. A theoretical exploration upon reciprocity in eldercare is hereby provided. These findings could shape improvements in aging and health care policies that are person-centered and focus on reciprocity.

Norway provides some of the best conditions for the aging, efforts which are supported by financial resources directed to pension funds and the health care system (Organization for Economic Co-operation and Development [OECD], 2014). However, isolation and loneliness remain some of the most influential causes of poor health and depression among older adults in the country (Samfunskunnskap, n.d.). Research is needed in order to understand the experiences of older adults, as on the basis of such research we could develop effective and inclusive interventions (Hauge & Kirkevold, 2010; OECD, 2014; Samfunskunnskap, n.d.). On the one hand, such an effective inclusion could be a way to lessen isolation and loneliness, as well as their side effects for the mental and physical health among older adults. On the other hand, recent findings suggest that in order to promote sense of community among older adults, it is crucial for them to perceive reciprocity in their relationships, and that they are still able to influence their communities (Bahl, 2018). Thus, interventions could stand to uncover some of the resources that the older adults already have, instead of merely focusing on their needs as care receivers (Dale, Söderhamn, & Söderhamn, 2012). This orientation could lead researchers to influence aging policies, addressing caring relationships in terms of their potential for health-promoting reciprocity, not just in terms of their implications for the national economy (Draper, 2014; Vernooij-Dassen, Leatherman, & Olde-Rikkert, 2011). However, the notion of reciprocity can appear as ambiguous in the literature (Lehmann, 2018a; Lewinter, 2003). Therefore, shedding light into it could support the effective implementation and assessment of community interventions among older adults. Precisely, in this article we address the notion of reciprocity theoretically, while documenting the way in which community interventions such as writing courses can promote it.

## Towards a reciprocity of care

Ideally speaking, developing reciprocity in healthcare is a collaborative process whereby patients, family members and healthcare professionals actively participate in communication and decisions (Vivian & Wilcox, 2000). Indeed, imbalances or ambiguities in the experience of reciprocity can fracture relationships (Neufeld & Harrison, 1995). However, most research on reciprocity is based on theories of social exchange and equity, focusing on family members and nurses as the main care providers, and seldom treating older adults as the primary focus of investigation (Hsu & Shyu, 2003; Neufeld & Harrison, 1995; Wilson, Morse, & Penrod, 1998).

Promoting experiences of reciprocity can improve mental health among older adults. In some cultures, maintaining a sense of independence as well as a sense of reciprocity between the older adults and health care professionals is a core value (Fyrand, 2010). This is the case for Norwegian culture, which promotes responsibility towards self-care practices, reducing the expectations that family members will act as care providers (Bahl, Nafstad, Blakar, & Geirdal, 2017). Yet, the vulnerability of older adults can make them susceptible to abuse and neglect, which threatens the experience of reciprocity (Lewinter, 2003). Thus, acknowledging the coexistence of degrees of independence and vulnerability could advance our understanding of reciprocity in health care relationships (Wilson et al., 1998). Doing so could also influence educational programs, since palliative care and eldercare research highlight the need to include reciprocity in the formal education of health care professionals (Janssen & MacLeod, 2012; Vernooij-Dassen, Leatherman, & Olde-Rikkert, 2011).

In this article, we theoretically elaborate upon reciprocity by analyzing narratives from the writing courses for older adults arranged by the Church City Mission in Oslo (here onwards CCM). This is a program financed by the Ministry of Health and Care Services in Norway, consisting of 10 weekly encounters each semester of the year. The classes last one and a half hours each, and they begin with the writers-teachers presenting a prompt, such as garden, dream or hand. The selection of these prompts is intuitive, aimed at concreteness, and can consist of single words, sentences or pictures. After the prompt, the participants speak about the topic for a while and then write about it on an individual basis for half an hour. Sometimes the writers-teachers write on behalf of some of the course participants who dictate their words. Next, the course participants read out loud what they have written and share their impressions with one another. The writers-teachers also give feedback about the texts, such as reflections or feelings that could be further elaborated (Ellenes & Bronke Senderud, 2015).

## Methodology

### “I´m the one who has written this”: a book as a portrait of human experience

We have analyzed the anthology “Det er jeg som har skrevet det” (I´m the one who has written this), which is divided into two sections. The section “thoughts” comprises 8 chapters written by writers who taught the courses. The writers-teachers included personal reflections on their experiences facilitating the writing courses, as well as observations and lived-experiences from the older adults who participated in them. Thus, in the data analysis, this section of the book is prioritized. The section “texts” comprises 59 short texts, which were written by some of the older adults who attended the courses in Oslo between 2009 and 2015.

This anthology is both a piece of literature and a journal. The analysis of literature has been crucial for the development of psychological theory (e.g., Vygotsky, 1925/1971), since accurate descriptions of phenomena in these works evoke the tensions and contradictions of human existence. They can also serve as a research account that synthetizes and crafts the reality of human everyday life in ways that classical research reports hardly convey (Brinkmann, 2012). This anthology portrays the efforts of the writers-teachers to interpret the significance of the encounters that took place during the writing course, but it also serves as a platform for journaling, both for the writers-teachers and for the course participants. Diaries have also been crucial for the development of psychological theory, especially in case study research (Allport, 1942). This is so because they are a tool for self-exploration that conveys the dialogical quality of the psyche (Lehmann, 2018b). That is, written accounts are also externalized signs of mental and developmental processes, which can be used to study the dynamics of meaning-making and affective processing and its social guidance.

### Technique of analysis

We used narrative analysis in order to identify the storylines in the texts and the tensions within individual and collective narratives (Josephson & Alsaker, 2015). This allowed us to remain open and reflexive when looking at the data, while also acknowledging how our theoretical backgrounds would guide the analysis (Murray, 2015).

### Procedure

The first author of this article manually selected 121 excerpts in the section “thoughts,” and 88 in the section “texts”, which summarized the contents of the book (this being the selection criterion). Then, we assigned preliminary codes to synthetize the storylines in each each excerpt, such as “life transitions”, “fears”, “expectations”, “rooms for affect”, “relationships” or “sense of otherness”. We analyzed the narratives in Norwegian and translated into English the excerpts presented in this article. During and after the transcription of the excerpts and notes to Excel and NVIVO files, the preliminary codes were reorganized into the following storylines: (a) relational movements reciprocity; (b) life journeys: The older adults´ portraits of their lives; (c) Existential meaning-making: deepening into our human condition; (d) motivation and purpose; (e) memory and attention. E-mail follow-ups with the writers-teachers and the coordinators of the project were also made in order to clarify information.

### Ethical considerations

The book analyzed in this article is a public document. Thus, our research did not require approval from ethical committees in Norway. We shared a preliminary version of this article with CCM, and have been given permission to use excerpts from the book. This research received no grant from any funding agency in the public, commercial, or not-for-profit sectors.

## Findings and discussion

### Relational movements reciprocity

**Figure 1.**
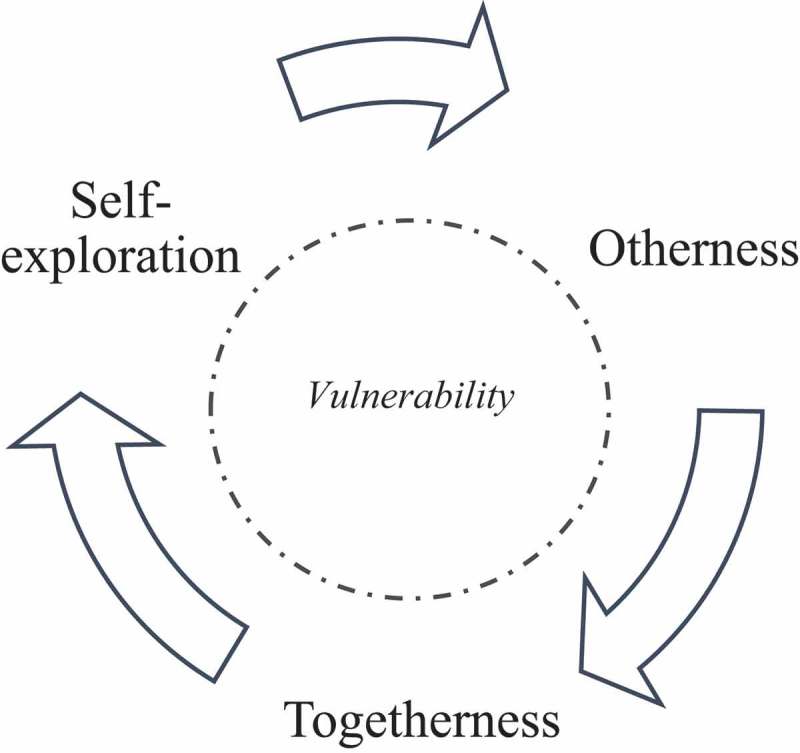
Relational movements of reciprocity.

The main storylines depicted in the book evoke three relational movements: self-exploration, otherness and togetherness, as we illustrate in Figure 1. These directions of reciprocity involve dialogical processes of relating to oneself<->oneself, oneself<->other, oneself<->togetherness, other<->togetherness. In addition, vulnerability appeared at the core of these relational movements, shaping the meetings between the “writers-teachers” and the “participants” in the plurality of their identities. Therefore, we place vulnerability at the center of the figure within dashed lines, suggesting a container for such vulnerability to be expressed. For example, it can be challenging for the writers-teachers to enter a space where they can meet their own vulnerabilities or to imagine meeting the vulnerabilities of their students:

The scariest thing I could imagine was trying to relate to people who need help with all that which one takes for granted when one is not dependent on care—life situations in which people may experience extra vulnerability and inadequacy. There is another level of adequacy experienced in the writing courses for older adults and there is something recognizable about it. But there is also a sort of vulnerability that one can either protect oneself from or decide to embrace. I believe that writing is a way of challenging one´s vulnerability. (Gabrielsen, 2015, p. 53)

The vulnerability experienced by older adults can be seen as threatening to caregivers and even if it can lead to neglect and abuse (Lewinter, 2003). Yet, in the writing-courses there was a deeper embrace of vulnerability as a part of the human condition. This opened the door for reciprocity to emerge, as these courses appeared to be transformational not just for the older adults, but also for the writers-teachers and even for some family members of both parties. Our acknowledgment of such reciprocity was understood by employees of CCM as a reassurance of their mission; that of working towards person-centered care practices (H. W. Kristiansen, personal communication, 24 September 2018). Norway, along with other countries, has recognized the need in health care services to ensure that services are developed in partnership with both the people served and the people providing the care, and the need to do so in a manner that is sensitive to context-specific needs (Vabø, Elstad, Drange, & Norvoll, n.d.).

#### Self-exploration: I see you and I see myself

Writing can aid the externalization of thoughts and feelings, and their further integration and internalization in new ways. Be it the possibility for understanding different positionings of the self, or gaining perspective on past, present or future relationships with others, self-exploration is a relational process (Lehmann, 2018b). At the writing courses, both writing and sharing what one has written offered the possibility to mirror and deepen self-exploration. For example, one participant, when asked how he/she experiences the course, wrote that: “The recognition of having delivered a piece of work gives me a boost—the feedback means a lot to me since it tells me something about the text I have written, which I do not see myself” (Ellenes & Bronke Senderud, 2015, p. 78). Yet, what the narratives from the writing course highlight is the fact that this process of self-exploration also appears on the teachers´ side:

Being a teacher. It should be the easiest thing in the world. But it has not been so for me. I still have days in which I find it hard (…) I look at myself as an involuntary teacher. I never wanted to be a teacher (…) There are goals. And there are funds. For many, the road to becoming a writer is paved with side jobs (…) (Ellenes, 2015, pp. 91–92)

Thus, the course becomes an opportunity for meaning-making, as facilitated by the act of writing and reflecting upon what one has written, as well as by verbally sharing with others. This possibility is also extensive within the interactions between course participants:

I especially remember when a participant who is quite modest opened up a lot, expressing that this is a place one can feel safe. I remember how surprised I was when I wrote about the time I lived alone as a teenager, while my mother was in the hospital. I did not think I was so emotionally affected by that experience. (Ellenes & Bronke Senderud, 2015, p. 86)

That is, the sense of reciprocity among writers-teachers and participants, as well as between the participants themselves, supports the process of self-exploration by the members of the writing course. This can facilitate creativity and meaning-making in relation to both experience and existence. At the same time, the purpose of the course is not limited to that alone. It is also the possibility to see the other, as reciprocity is one of the conditions to fully express the ethical aspects of care and otherness (Freeman, 2017). That is, narratives are dialogical both inside the perimeters of the self, in the form of I-me relationships, but also outside the self, in terms of otherness (Freeman, 2017).

#### Otherness: I see you and I feel seen

This relational movement appears as the intention to see the other as human, as seeing the other in their uniqueness that transcends the labels of either being “writers,” “teachers” or “older adults,” and as recognizing feeling seen by the other. One participant speaks to these intentions when sharing these words with the course facilitators:

I very much like to listen to the texts of others. I admire the wording, the memories and the imagination. I admire the humor. There is laughter during the class. Sometimes it is also touching to listen to what the others write. They often open themselves up to their innermost rooms. It is so good. Because of this, I also understand how different we are as human beings. (Ellenes & Bronke Senderud, 2015, p. 81)

Our relationships with others shape our narratives and this recognition of the other is inherent to, and sometimes even prevalent over, the understanding of a singular self. In order to illustrate his theoretical arguments concerning otherness, Freeman (2016, 2017) speaks of his personal experiences visiting his mother who had dementia. He focuses not just on the recognition of the other, but also on caring for the other, recalling Levinas´ ideas regarding responsibility towards the other, as well as Buber´s understanding of I-thou encounters, which imply a sense of reciprocity. From the perspective of narrative care, reciprocity is closely related to the sense of otherness, and attempts at respecting the other, preserving their stories, and caring for his/her humanness (Freeman, 2016). As the data suggest, the sense of otherness is embraced as a process of moving the focus of attention and intention outside of the self, be it by the writers-teachers towards the participants, by the participants towards other participants, or by the participants towards the writers-teachers. This relational movement, we hereby suggest, is necessary for the experience of encounter. An encounter, or genuine dialogue, implies that human beings feel seen and see each other as a whole, and allow themselves to be transformed as individuals, as a community or as a society (Buber, 1939/1950; Cooper, Chak, Cornish, & Gillespie, 2013). Experiencing an encounter involves the recognition of the uniqueness of human existence, both one’s own and those of others. In the following excerpt, one of the writers-teachers illustrates the human fibers that are interwoven in the encounter as a reciprocal act:

“So, is it fine that I write about you? What do you write about then?” She asks. About the fact that you write! (…) I write about some of the experiences you have had and to which you constantly return in your texts (…) She is one of the many course participants who does not care about age. I am not someone who is 50 years younger and does not understand, and she does not give herself the role of one who is old and outdated–no, we meet in conversations that emerge about the compositional-work, not in a generational gap. Because I also continue to work with texts long after I have left the room. Several participants of the course say that once they come to their own rooms, they remember other things they will include in their texts (Gabrielsen, 2015, pp. 66–67)

This quote reflects reciprocity in full blossom, highlighting the transformational space that writing courses can open up for both their writers-teachers and their participants. This experience of human connection appears to become motivational, and yet, it also transcends the experience of otherness, inviting us to explore a further relational movement, that of togetherness.

#### Togetherness: we see ourselves

The third relational movement present in the narratives is togetherness. This movement involves a perception of “we” as a focus of attention and intention in the narratives. This can give account of a psychological sense of community, which implies a sense of belonging, mutual influence, fulfillment of needs and shared emotional connection (McMillan & Chavis, 1986). A writer-teacher beautifully recalls this when saying:

As often occurs during the writing courses, I once again got the strong feeling that age does not prevent us from the most important thing. Whatever it may be … maybe it is being able to meet another person with respect because both of you have something to give to each other (…) I would rather say that we met in a shared passion for writing, hungry to continue talking about texts and all the places that the texts suggest or open up for us, even after the writing course. (Gabrielsen, 2015, p. 59)

In addition, one of the values that is crucial for the sense of “we” is trust, as another writer-teacher highlights when saying that:

To write in a group is in many ways about trust. This is something we consciously work to build up, and to be safe in the group is as important as it is to trust the words that emerge, that nothing is too small, wrong or silly. (Bronke Senderud, 2015, p. 39)

Developing reciprocal trust is the basic process underlying nurturing relationships among care-givers and care-receivers (Wilson et al., 1998). Given the conditions of trust, openness and vulnerability that give room for reciprocity to unfold--in any variety of constellations (e.g., oneself-oneself, oneself-other, or togetherness)--, these writing courses becomes a catalyst for meaning-making. In the next subsections we extend upon this.

### Life journeys: the older adults’ portraits of their lives

Whether or not the texts produced by the course participants are based on creative writing or autobiographical accounts, they often wrote about their past, even if it was not mandatory to for them to write about their lives. That is, the writers-teachers focused on giving feedback on the texts, instead of discussing the veracity of the stories that are being narrated. The older adults described a wide range of human experiences. The texts became an opportunity for remembrance and an opportunity to relate with significant others and with themselves, since most of the narratives referred to family, romantic relationships, and fortunes and misfortunes. Their stories were about diverse life transitions such as moving between rural or urban areas, traveling, and migrating. One of the main storylines that appeared was life through the landscape of war. Yet, the plots of their stories were not necessarily about war, but about their life trajectories unfolding besides the war. This quotation exemplifies these different topics coming together, even if in other cases the themes were presented separately by different persons:

The first place that I remember is a small house. I was five years old at the time. During the war. I went for a walk by myself. To a stream. There was a German who blew up a balloon. The first delicate and blue balloon of my life. I was so proud. But when I went back home, dad was standing at the stairs and looking at me. The balloon fell out of my hands into a bush. Pang, it sounded. “Come in!” Said dad. (Egg, 2015, pp. 38–39)

Previous research based on diaries written during the World War II provides insights into how “people move through complex worlds, and how experiences from distal contexts interact with immediate experiences, becoming more or less integrated” (Zittoun & Gillespie, 2015, p. 41). That is, by looking back in time towards aspects of one´s biography, one can see how memories and imagination are shaped by the context in which they originally occurred. One can also see how they can transcend that context and can gain new meanings in the present by shifting attention to different aspects of a narrative, such as one´s first balloon instead of the surrounding war. Another aspect of such reconstructions of the past enabled by writing in the courses is the possibility of expressing life journeys as a composition of both events that occurred and imagined futures that never manifested themselves. In doing so, the older adults are unveiling a wisdom of our human condition, that of embracing the uncertainty of life and that which escapes of the reach of our longings. A participant exemplifies this tension:

I have no children. This is a story with a double meaning. I was engaged to a Swede, but he died just 14 days before our wedding (…) One time, the husband of my girlfriend said: “Can I bring a friend with me?” (…) We met each other at 54 and married at 59 (…) I had a good marriage”. (Gundersen, 2015, p. 64)

Life trajectories are influenced by shadow trajectories, which are imagined realities that do not manifest themselves, but the non-occurrence of which shapes the occurrence of other decisions and events (Bastos, 2017). By giving account of these imagined futures, some participants tried to give meaning to their lives, or to accept the uncertainty of that which did not happened. Narratives in themselves become an effort to justify events, to make sense of uncertainty (Bruner, 1990) and the writing courses provided older adults with this possibility. As many older adults need such a possibility, this element of the writing courses is of great value to therapeutic or community interventions.

However, the course participants did not always elaborated upon the emotional implications of their experiences. In a follow-up with one of the writers-teachers, she suggests that this is so since: “We are not therapists but fiction writers (…) even if the starting point is not to do a therapeutic work, it is often found that writing courses can have a therapeutic effect (H. Aanestad, personal communication, 24 August 2018). Indeed, even if these courses were not framed as being therapeutic, their therapeutic implications should not be underestimated, since writing can improve physical and mental health (Wright & Chung, 2001). Still, the therapeutic effects of writing are catalyzed by writing about emotions, even those that can be traumatic (Pennebaker & Seagal, 1999). For instance, one course participant was able to grieve while writing at the course, suggesting the therapeutic potential of these interventions. On day, she could not write further about the prompt “hands,” since it reminded her of her son who had drowned. After this, a writer-teacher noted: “We tried to put tasks that did not trigger their greatest sorrows/grieves” (Bronke Senderud, 2015, pp. 37–38), yet those topics emerged later. The writer-teacher describes it as follows:

So, one day, after many semesters, yes, many years, we had a task, I think it was either “dream” or “stream” (…) She sat down and wrote the whole time she had at her disposal (…) she read word by word, and all of us who had been sitting together week after week over many years were deeply moved, because we sensed the loss, the pain, but also the joy for the words having found their way out, so that it was possible to write. Write and share. (Bronke Senderud, 2015, pp. 37–38)

Our life journeys involve challenging experiences, such as relating with the death of others, or our own death, and writing can be an opportunity to make sense of that which is ineffable about life itself, an opportunity for existential meaning-making, as we expand in the next section.

### Existential meaning-making: deepening into our human condition

In order to promote well-being among older adults, it is crucial to provide support to existential challenges that aging involves (Schulz & Monin, 2018; Shaw, West, Hagger, & Holland, 2016). The writing courses developed by CCM are a platform for existential meaning-making, even if this is not a specific goal in the class, as one writer-teacher exemplifies when saying that: “We know that many of the texts come from their own lives, but we seldom speak about it, we limit ourselves to the texts themselves (Ramsdal, 2015a, p. 27).” Even so, writing in the context of a class might promote self-exploration by means of questioning one´s own existence (Lehmann, 2018b). This represents some challenges, since:

It is challenging when people have to finish the writing course due to poor health, relocating to another nursing home (…) It may be challenging as a writing teacher to give room to very different things: participants who are in contact with strong feelings. Others who feel uneasy or inadequate, someone who has a break-through when writing. (Aanestad, personal communication, 24 August 2018)

In addition, the narratives of the older adults at times evoked philosophical inquiry. For instance, one participant wrote: “Abortion. Is it immoral? (…) No, I don´t know, moral and immoral (…) But we are just humans” (Mortensen, 2015, pp. 66–67). At times, their stories referred to the ephemeral and complex character of life, including regrets, uncertainty and even the feeling that some aspects of their life-stories remained unfinished. These reflections upon the impermanence of life can nourish the understanding of purpose in life (Kübler-Ross & Kressler, 2000). In addition, the writers-teachers themselves reflected upon the possibility of explicitly bringing in existential topics, yet this evoked some ambivalence, reflecting their own personal vulnerabilities:

I think that one of the next times I will ask them about death. Have it as a topic. Maybe calling it “birth and death,” since the topic is more related to the life-cycle than just death (…) I have wondered how to bring up the topic. How to talk about it without becoming too heavy. I told them about my mother who died of cancer 10 years ago. (Ramsdal, 2015b, pp. 114–116)

When introducing dilemmatic topics which touch upon existential questions, such as death, the writers-teachers made two methodological choices. First, they presented death as a theme by disclosing a personal story that modeled the vulnerability and humanity in which participant stories could be framed. The idea of nearness to death and anxiety regarding the end-of-life are common even in healthy older adults (Bergman, Bodner, & Shrira, 2017), and by giving room to these topics, the vulnerability and reciprocity embraced at these courses evokes the human condition. The second methodological choice was that of introducing dilemmatic topics in terms of polarities in tension. Contradictory feelings (e.g., feeling happy to be alive, yet tired of physical detriment) are common in eldercare (Hvalvik & Reierson, 2015; Lehmann, 2018b). Yet, there are seldom spaces to work this through in this context. The coexistence of these polarities in tension might enable participants to embrace the complexity of affective processes, while they are finding meaning to their experiences or to their existence. One possibility for further exploration, about which the data do not speak, is the use of poetry to encourage deeper exploration of these tensions. Research suggests that doing so can aid existential meaning-making, bringing life into perspective, and enabling people to connect with the beauty of life, even in its tragic nature (Lehmann, 2018b). For example, co-constructed poetic accounts of the life-stories of people in palliative care have the potential for identifying crucial needs and wants among the participants (Synnes, 2015, 2016). Using poetry, among other tools, life´s fragility and finitude can gain a sense of beauty and sacredness that expands our understanding of the ethics of care, and of our human condition (Freeman, 2016). For example, when referring to a 92-year-old patient, Siegel (2010) reflects upon their therapeutic process of finding words to depict the complexity of emotional experiences. The gift of poetry, Siegel follows, is its skillful embrace of words, which most of us human beings could learn from.

### Motivation and purpose

In connection with existential meaning, the writing courses had a motivational impact on both the course participants and the writers-teachers. Perhaps it is never too late to learn something new, as one of the participants of CCM´s writing courses expressed: “It was a completely new experience for one who is 91 years old, something new and exciting” (Ellenes & Bronke Senderud, 2015, p. 83). Indeed, some of the participants continue to work on the texts after the class is over, either because they continue to remember or to process that which the prompt or the conversations elicited, or because they feel motivated to write in their free time. In addition, the texts that the older adults write might not only serve them, other course participants or the writers-teachers. They also have an impact in other systems, such the family ones:

Often, the texts are read at their funerals and their family says they got to know other sides of their grandmother or their uncle, and I have been wondering which kind of texts would my grandfather have written if he would have been part of a writing course at his nursing home. How would it have changed the way I see him? (Ramsdal, 2015a, p. 22)

That is, the wide canvas of wisdom that the older adults have about life, and the ways in which they depict moral values, history, real stories, or even fiction, could have a social impact if shared with different communities. Precisely, this is one of the aims of the Legacy Project (Pillemer, n.d.), where older adults are interviewed about lessons from their life-stories which can inspire others; legacy that could be considered a form of reciprocity. This could be so, understood as the potential to reciprocate care in the future, be it to the person who is taking care of them in the present, or to someone else (Neufeld & Harrison, 1995). Emphasizing the impact that the stories of the older adults have in the community could motivate them to share their stories to a wider scale, even if they will not get to know in person those who might be inspired or entertained by their words. Thus, both storytelling and writing can have motivational effects in older adults, promoting the sense of purpose in their lives.

Furthermore, the capacity to explore oneself and touch one´s own human condition, to embrace a shared sense of humanity, to recognize the other, and to embrace the togetherness that is created in the writing courses, was felt as motivational. This challenges some statements which highlight the importance of reciprocity, but at the same time treat older adults as frail, passive and vulnerable, which of course affects the experiences and expressions of reciprocity in themselves (Lewinter, 2003; Vernooij-Dassen, Leatherman, & Olde-Rikkert, 2011). Treating others as human beings, beyond considering them as mere recipients of care, encourages a sense of independence and builds self-esteem (Wilson et al., 1998). These implications could be extended to eldercare, since some of the needs and wants of older adults at the end of their lives relate to the reciprocity of giving and receiving, the experience of belonging, agency, and gratitude for life in general (Synnes, 2015, 2016).

### Memory and attention

One of the most salient themes described by the writers-teachers when referring to their course participants were memory and implicitly, attention. The writers-teachers referred to memory as an embodied process that is contained by the possibilities and limits of language (Gabrielsen, 2015). In doing so, they recalled aspects of lucidity, remembrance and forgetting that they considered meaningful during the course, even in cases of dementia. For example:

We had a female course participant who became increasingly passive as her dementia evolved. (…) she understood us when we introduced the writing task of the day, for short and poetic texts always emerged from her, conveying the task´s theme. Yet, she eventually withdrew and remembered nothing, she said (…) at least for this woman, touching the body was closely related to language. Was it memory which was coming to life? I remember that the writing task was about her husband, so it felt natural for me to touch her wedding ring. Inside her wife´s body their life together hasn´t been forgotten, it was just hiding itself in strange places and periods, but writing could lock it out and bring it to life. (Gabrielsen, 2015, p. 56)

Writing courses appear to be a socially inclusive practice that could benefit the mental and physical health of older adults in general, and persons who suffer from dementia in particular. Storylines such as the one presented in the quote above are suggestive of the possible role of the writing courses in improving memory and attention. These implications require further investigation, other than the perceptions of the teachers, and the experiences of course participants. Further psychological research that would test the improvement of memory and attention could be of relevance here, especially since these courses are funded by the Norwegian Ministry of Health and Care. Analyzing the effects of using poetry among other literary forms could be further emphasized as well, given its capacity to evoke powerful emotional experiences. This is so since poetry is the most musical realm of language, and the effects of music in interventions for dementia have already been studied (Gerdner, 1999, 2005). It is also possible that the experiences of reciprocity catalyze such effects since other researchers have found reciprocity to be a key mediator in the success of community or occupational therapy interventions in dementia (Vernooij-Dassen, Leatherman & Olde-Rikkert, 2011). Salutogenic initiatives such as this also relate to reciprocity, since health care professionals could focus their attention in order to monitor signals of reciprocation of care, however subtle (Neufeld & Harrison, 1995; Vernooij-Dassen & Moniz-Cook, 2016). Among care-receivers and care-givers, the perception of reciprocity could be enhanced by modeling trust in the other as a person, being willing to negotiate during interactions or by acknowledging hints of attentional shifts, such as posture, humor, touch, or enjoyment (Wilson et al., 1998).

## Conclusion

In this article, we provided a theoretical understanding of reciprocity of care, by means of highlighting the relational movements experienced during the writing courses for older adults that CCM coordinates in Norway. That is, both writers-teachers and course participants relate to each other and to themselves through the lenses of self-exploration, otherness and togetherness, which are co-constructed and maintained throughout the writing courses. By treating reciprocity as a process, rather than a static concept, we are opening the path for further conceptualizations that enable treating older adults and caregivers as active agents. However, reciprocity is neither explicitly promoted nor naturally developed in all caring relationships. Thus, further educating not just the facilitators of writing-courses, but also health care professionals, can improve caring practices by focusing on developing reciprocity. This outline could influence the ways in which aging is addressed by policy-makers and the ways it is addressed within the education of health care professionals and teachers.

In connection with this, the relational movements of reciprocity in CCM´s writing courses appeared to be shaped by the sense of vulnerability that both writers-teachers and participants described as necessary to co-create a space of belonging that could be beneficial for motivation, memory and attention, even if more consistent evidence upon the impact on these psychological functions is needed. If proven to effect motivation, memory and attention, writing courses could be implemented as strategies of mental health promotion, on top of being effective community interventions to prevent loneliness, depression or even suicide attempts among older adults. Further research integrating interdisciplinary approaches with psychology could assess the impact of these writing courses on mental health. Even more, the motivational benefits can be extensive for the writers-teachers, for family members and for the community in general—and all the more so by the publication of the book.

In addition, writings courses are cultural tools that mediate the reciprocity of care practices and, even if not being explicitly therapeutic but rather pedagogical and cultural, they can have therapeutic effects. Thus, collaborating with psychologists in developing such courses could help further afford older adults the opportunity to explicitly elaborate on complex topics such as death, or grief. In exploring complex affective processes, poetry could be also explored as a tool to embrace the tensions that form emotional experiences, especially in relation to existential meaning-making.

Given the fact that these courses are already financed by the Norwegian government, it would be important that the outcomes of writing courses alike could also shape improvements in aging and health care policies. A possibility to do so, which is to be considered in further research projects, is to bridge the notion of reciprocity with theories such as person-centered care. This could enhance the coherence with the ways in which the Norwegian government wants their health care services to develop (Norwegian Ministry of Health and Care Services, 2015). These person-centered care practices might also involve a focus on the inclusion of minorities, since writing courses might presuppose being fluent in Norwegian languages. Future courses might either provide the possibility for older adults from different cultural backgrounds to write in their mother-languages or they might place the focus on story-telling and not just on writing. Indeed, the courses of CCM could be considered as both a writing and storytelling setting, given that the course participants also verbally share their experiences. CCM is currently trying to make their writing courses more inclusive in this regard.
